# Liquid biopsy reveals KLK3 mRNA as a prognostic marker for progression free survival in patients with metastatic castration‐resistant prostate cancer undergoing first‐line abiraterone acetate and prednisone treatment

**DOI:** 10.1002/1878-0261.12933

**Published:** 2021-05-29

**Authors:** Emmy Boerrigter, Guillemette E. Benoist, Inge M. van Oort, Gerald W. Verhaegh, Onno van Hooij, Levi Groen, Frank Smit, Irma M. Oving, Pieter de Mol, Tineke J. Smilde, Diederik M. Somford, Niven Mehra, Jack A. Schalken, Nielka P. van Erp

**Affiliations:** ^1^ Department of Pharmacy Radboud University Medical Center Radboud Institute for Health Sciences Nijmegen the Netherlands; ^2^ Department of Urology Radboud University Medical Center Radboud Institute for Molecular Life Sciences Nijmegen the Netherlands; ^3^ MDxHealth Nijmegen the Netherlands; ^4^ Department of Medical Oncology Ziekenhuisgroep Twente Almelo the Netherlands; ^5^ Department of Medical Oncology Gelderse Vallei Hospital Ede the Netherlands; ^6^ Department of Medical Oncology Jeroen Bosch Hospital ‘s Hertogenbosch the Netherlands; ^7^ Department of Urology Canisius Wilhelmina Hospital Nijmegen the Netherlands; ^8^ Deparment of Medical Oncology Radboud University Medical Center Nijmegen the Netherlands

**Keywords:** abiraterone acetate, biomarkers, Castration‐resistant prostate cancer, *KLK3*, liquid biopsy, RNAs

## Abstract

Circulating RNAs extracted from liquid biopsies represent a promising source of cancer‐ and therapy‐related biomarkers. We screened whole blood from patients with metastatic castration‐resistant prostate cancer (mCRPC) following their first‐line treatment with abiraterone acetate and prednisone (AA‐P) to identify circulating RNAs that may correlate with progression‐free survival (PFS). In a prospective multicenter observational study, 53 patients with mCRPC were included after they started first‐line AA‐P treatment. Blood was drawn at baseline, 1, 3, and 6 months after treatment initiation. The levels of predefined circulating RNAs earlier identified as being upregulated in patients with mCRPC (e.g., microRNAs, long noncoding RNAs, and mRNAs), were analyzed. Uni‐ and multivariable Cox regression and Kaplan–Meier analyses were used to analyze the prognostic value of the various circulating RNAs for PFS along treatment. Detectable levels of kallikrein‐related peptidase 3 (*KLK3*) mRNA at baseline were demonstrated to be an independent prognostic marker for PFS (201 vs 501 days, *P* = 0.00054). Three months after AA‐P treatment initiation, *KLK3* could not be detected in the blood of responding patients, but was still detectable in 56% of the patients with early progression. Our study confirmed that *KLK3* mRNA detection in whole blood is an independent prognostic marker in mCRPC patients receiving AA‐P treatment. Furthermore, the levels of circulating *KLK3* mRNA in patients receiving AA‐P treatment might reflect treatment response or early signs of progression.

## Graphical abstract

Circulating RNAs extracted from liquid biopsies represent a promising source of cancer‐ and therapy‐related biomarkers. *KLK3* mRNA detection in whole blood is an independent prognostic marker for progression‐free survival in mCRPC patients. *KLK3* mRNA levels probably correspond to the number of circulating tumor cells. Therefore, *KLK3* detection may be a potential biomarker to monitor treatment response but prospective clinical validation is needed.

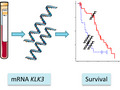

**Abbreviations**
AA‐P: abiraterone acetate and prednisone
AR‐V7: androgen receptor splice variant 7
CI: confidence interval
Cp: crossing point
CTC: circulating tumor cell
*C*_trough_
: concentration before the next dose
CV%: coefficient of variation
Hb: hemoglobin
HR: hazard ratio
*KLK3*
: kallikrein‐related peptidase 3
KM: Kaplan–Meier
LDH: lactate dehydrogenase
LLN: lower limits of laboratory normal
lncRNA: long noncoding RNA
mCRPC: metastatic castration‐resistant prostate cancer
miR‐141: microRNA 141
miR‐200a: microRNA 200a
miR‐200c: microRNA 200c
miR‐21: microRNA 21
miR‐3687: microRNA 3687
miR‐375: microRNA 375
miRNA: microRNA
ncRNA: noncoding RNA
OS: overall survival
PBMCs: peripheral blood mononuclear cells
PCa: prostate cancer
*PCA3*
: prostate cancer‐associated 3
PCWG: Prostate Cancer Working Group
PFS: progression‐free survival
PSA: prostate‐specific antigen
qPCR: quantitative polymerase chain reaction
*SCHLAP1*
: SWI/SNG antagonist associated with prostate cancer 1
SL‐RT: stem‐loop reverse transcriptase
*TMPRSS2‐ERG*
: transmembrane serine protease 2‐ETS transcription factor ERG fusion gene product
ULN: upper limit of normal

## 1 Introduction

Prostate cancer (PCa) is the second most common cancer in men worldwide [1]. It presents with a wide range of disease stages, from localized PCa to lethal metastatic castration‐resistant prostate cancer (mCRPC). The therapeutic landscape for patients with mCRPC is rapidly evolving, with several new therapies improving overall survival (OS). Abiraterone acetate and prednisone (AA‐P) is one of these therapies that has been proven to prolong OS in patients with mCRPC [2, 3]. However, suboptimal response [*de novo* resistance or shorter progression‐free survival (PFS)] has been observed in a subset of patients and eventually all patients develop therapy resistance. To improve outcome and cost‐effectiveness, it is important to select patients for treatment with AA‐P that will benefit most.

Liquid biopsies are a promising source for biomarker analysis. Besides circulating tumor DNA and circulating tumor cells (CTC), liquid biopsies also contain circulating RNAs, protein‐coding mRNAs, and noncoding RNAs (ncRNAs), such as microRNAs (miRNAs) and long noncoding RNAs (lncRNAs), all of which can be released by the tumor into the circulation [4]. Liquid biopsies are minimally invasive, and therefore, longitudinal sample collection and biomarker analysis are feasible. Furthermore, with liquid biopsies it is possible to identify tumor heterogeneity in a single biopsy [5].

Detection of cancer‐specific mRNAs has the potential to characterize the tumor and determine tumor burden in blood. The prostate‐specific *kallikrein‐related peptidase 3* (*KLK3*) mRNA, which codes for prostate‐specific antigen (PSA), is a clinically validated diagnostic marker used in urine‐based tests [6]. Furthermore, the predictive value of *KLK3* mRNA decreases in patients with advanced PCa treated with docetaxel has been explored [7]. Danila *et al*. [8] have developed and validated a droplet digital polymerase chain reaction (ddPCR)‐based assay to detect gene transcripts (*KLK2*, *KLK3*, *HOXB13*, *GRHL2,* and *FOXA1*) that are highly expressed in prostate tissue and peripheral blood from patients with mCRPC. The AdnaTest is an assay that detects *KLK3*, *PSMA,* and *EGFR* transcripts in CTCs captured on magnetic beads [9]. Both the AdnaTest and ddPCR are considered positive if at least one of the transcripts is detected. In a clinical validation study, Danila *et al*. [9] showed that *KLK3* detection was the primary marker for positive AdnaTest and ddPCR test results. Therefore, they suggested to remove the less‐useful transcripts from those tests and only measure *KLK3*. However, these results have not been confirmed in a prospective clinical trial yet.

MiRNAs are short (~ 21 nucleotide) single‐stranded RNAs that are able to drive tumor initiation and progression by controlling the expression of oncogenes and tumor suppressor genes [10]. Clinical studies showed that upregulation of miR‐21, miR‐141, miR‐200a, miR‐200c, miR‐375, and miR‐3687 are related to shorter PFS or OS in patients with CRPC [11, 12, 13].

LncRNAs are lncRNA transcripts with a length of more than 200 nucleotides [14]. An aberrant expression of many lncRNAs has been associated with PCa [15]. *Prostate cancer‐associated 3* (*PCA3*) is currently used in PCa diagnostic urine tests [16, 17]. Furthermore, the association of *PCA3* levels with treatment outcome was explored in a small cohort of patients treated with docetaxel [7]. Another lncRNA which has been shown to be upregulated in PCa tissue compared with benign tissue is *SWI/SNG complex antagonist associated with prostate cancer 1* (*SCHLAP1*) [18, 19]. Beside these, many other lncRNAs are being explored but the prognostic or predictive value for patients with mCRPC is yet unclear.

Although preclinical work revealed many biomarkers of potential use, the number of biomarkers that are translated to the clinical practice is very disappointing. In the biomarker landscape, many researchers are discovering new biomarkers, but without further validation their clinical usefulness is limited [20]. The ultimate goal is to implement biomarkers in clinical practice, and therefore, prospective validation is important and crucial. For AA‐P, only androgen receptor splice variant 7 (*AR‐V7*) is used as a predictive biomarker. Detection of *AR‐V7* in CTCs is associated with abiraterone acetate and enzalutamide resistance [21, 22]. Though, the detection and thereby use of *AR‐V7* as a predictive marker appears to be treatment line specific, since *AR‐V7* is only detectable in 3% of first‐line patients vs 18–31% in second line or higher and is therefore only useful to guide treatment in more advanced mCRPC patients [23]. Therefore, it also seems to be very important to study the value of biomarkers in a well‐defined stage of disease.

A prognostic index model for chemotherapy‐naïve patients treated with AA‐P has been described, including the following clinical parameters: presence of lymph node metastasis, lactate dehydrogenase (LDH) > upper limits of laboratory normal (ULN), ≥ 10 bone metastasis, hemoglobin (Hb) ≤ lower limits of laboratory normal (LLN), and PSA > 39.5 ng·mL^−1^ [24]. However, the additional value of novel biomarkers and drug exposure in this model has not been well‐studied. Abiraterone shows substantial interpatient variability in drug exposure [25]. For abiraterone, a minimum concentration (*C*_trough_) of 8.4 ng·mL^−1^ is suggested as a threshold for efficacy [26, 27]. Suboptimal exposure, defined as a *C*_trough_ below 8.4 ng·mL^−1^, has never been incorporated in prediction models before. Therefore, a prospective, observational multicenter study was conducted to explore the value of (pre)clinically identified promising circulating RNAs as prognostic biomarkers as well as the influence of abiraterone exposure on PFS in first‐line mCRPC patients (ClinicalTrials.gov ID: NCT02426333).

## 2 Materials and methods

### 2.1 Study design

This prospective, observational, multicenter study was conducted in five hospitals in the Netherlands. All patients with mCRPC starting first‐line AA‐P treatment, according to the drug label, were eligible. Patients were allowed to be pretreated with upfront docetaxel according to CHAARTED/STAMPEDE protocols in a hormone‐sensitive prostate cancer setting. Comedication that affected abiraterone pharmacokinetics was not allowed (e.g., potent CYP3A4 inhibitors and inducers). Patients were replaced if they stopped treatment or had dose reductions before the second visit (1 month after start). The study was conducted in accordance with Good Clinical Practice and the Declaration of Helsinki and approved by our Investigational Review board. Written informed consent was obtained from all patients before entering the study.

To identify biomarkers that are upregulated in PCa patients, blood from healthy individuals was used as control. Thirty healthy individuals (10 men < 35 years, 10 men between 55 and 70 years, and 10 women [no age restriction]), gave written informed consent for the use of an aliquot of their donated blood as control.

### 2.2 Assessments

During the study period of 6 months, patients had to visit the hospital at baseline, 1, 3, and 6 months after inclusion for physical examinations, laboratory tests, and blood collection. For biomarker analysis, blood was collected in PAXgene Blood RNA Tubes (PreAnalytiX; Qiagen/BD‐company, Hombrechtikon, Switzerland) at each visit. Plasma was collected in EDTA tubes at 1, 3, and 6 months for measuring abiraterone concentrations. Patients were instructed to take their abiraterone acetate (1000 mg once daily, combined with 10 mg of prednisolone) in the morning 1 h before breakfast. At the day of pharmacokinetic assessment, AA‐P was taken after the first blood collection for measuring the abiraterone trough level. Patients filled out a diary to check for drug adherence and side effects. Their diary and concomitant medication were checked during every visit.

Imaging was performed at baseline, 3, and 6 months after start of therapy. Tumor response was assessed by the treating physician and by an independent investigator during the study period, according to Response Evaluation Criteria in Solid Tumors (RECIST) v1.1 criteria. Progression during the study was defined according to the Prostate Cancer Working Group 3 (PCWG3) criteria. For patients not progressing before the final study visit at 6 months, progression thereafter was assessed by the treating physician. Survival data were collected from patients' medical record by the research nurse. Progression could be radiographic, biochemical, or clinically. Quality check of data‐entry was performed by an independent monitor.

### 2.3 Biomarker analysis

#### 2.3.1 RNA isolation from whole blood

The following biomarkers were selected for analyses based on previous data; mRNAs: *AR, AR‐V7*, and *KLK3*; miRNAs: miR‐21, miR‐141, miR‐200a, miR‐200c, miR‐375, and miR‐3687; lncRNAs: *AC012531.25, NAALADL2‐AS2, PCA3, SCHLAP1*, and *SNHG3* [7, 11, 12, 13, 18, 21, 22, 28, 29].

Total RNA was isolated from whole blood collected in PAXgene Blood RNA Tubes (blood volume 2.5 mL with 6.9 mL additive), using the PAXgene Blood miRNA and PAXgene Blood RNA kits (PreAnalytiX; Qiagen/BD‐company), according to the manufacturer’s instructions. The biological source of total RNA is both cells and exosomes. In this study, we included only first‐line patients, and hence, the number of CTCs is expected to be very low, and therefore, the isolated RNA is mainly from blood cells and tumor‐derived exosomes and only minimally from CTCs. (Cell free RNA is very unstable and susceptible to degradation, and will therefore not be detected.) PAXgene blood RNA tubes (Qiagen) are specifically developed and validated to isolate RNA from blood specimens. PAXgene tubes allow instant preservation of RNA, and the quality of RNA extracted using these tubes has been thoroughly investigated [30].

For miRNA analysis, each blood sample was first spiked with 2.0 fmoles of each *C. elegans* miR‐39 and miR‐238 (Table S1). RNA was eluted from the columns using 50–80 µL elution buffer. The samples were stored in nonstick RNase‐free tubes at −20 °C until further use. Total RNA quantification was performed on a Qubit 3.0 Fluorometer using the Qubit RNA BR Assay Kit (Thermo Fisher Scientific, Waltham, MA, USA). RNA quality was assessed on an Agilent 2100 Bioanalyzer Instrument using RNA Nano Chips (Agilent, Santa Clara, CA, USA). The median RNA integrity number value was 8.5 (range 5.4–9.9).

### 2.4 Reverse transcriptase and real‐time PCR analysis

Gene expression analysis was performed by relative quantification of mRNA levels and levels of the *PCA3* lncRNA using fluorescence‐based quantitative real‐time PCR assays, which were developed according to the MIQE guidelines [31]. Total RNA (extracted with the PAXgene Blood RNA Kit) was used for cDNA synthesis. RNA was first treated with DNaseI, and then, cDNA was synthesized, essentially as described by Dijkstra *et al*. [32]. RNA levels were determined by real‐time PCR using gene‐specific primers and hydrolysis probes (Tables S2,S3) and a LightCycler LC480 Instrument (Roche, Basel, Switzerland). Crossing point (Cp) values were calculated using the 2nd derivative method and the LightCycler LC480 Software (Roche). Copy numbers were calculated using calibration curves with a wide linear dynamic range, generated by serial dilutions (10–1 000 000 copies) of linearized plasmids (pCR2.1‐TOPO vector‐based) containing the target gene sequences.

MiRNA levels were measured using a stem‐loop reverse transcriptase (SL‐RT) PCR, adapted from Chen *et al*. [33]. SL‐RT was performed, using 2.0 µL of total RNA (extracted with the PAXgene Blood miRNA Kit) and 0.375 pmoles miRNA‐specific SL‐RT primer (Table S4). MiRNA levels were corrected for differences in blood volume. For lncRNA analysis, 250 ng total RNA (extracted with the PAXgene Blood RNA Kit) was used for cDNA synthesis. RNA was first treated with DNaseI, and then, random‐primed cDNA was synthesized using SuperScript II Reverse Transcriptase (Invitrogen, Carlsbad, CA, USA). RNA levels were determined by SYBR Green real‐time PCR using a LightCycler LC480 Instrument (Roche). Relative RNA levels were calculated using the weighted average of the *C. elegans* spiked‐in miRNAs (for miRNAs) or *GAPDH* (for lncRNAs) levels for normalization. Primer sequences are listed in Tables S2,S5; RT reaction conditions and PCR cycle conditions are listed in Tables S6,S7.

#### 2.4.1 Biomarker analysis in healthy controls

MiRNAs and the AR mRNAs were measured in 30 healthy individuals. LncRNA levels were measured in 10 age‐matched men. Levels of *KLK3* mRNA and *PCA3* lncRNA were not measured in healthy controls, since previous work revealed that these transcripts are only present in CRPC patients compared with healthy individuals [7]. Average Cp values in healthy controls were calculated and used as a reference.

#### 2.4.2 Biomarker analysis in patients

RT–PCR was performed twice for each sample to test for reproducibility. The RNA levels of *AR*, *AR‐V7*, *KLK3,* and *PCA3* were analyzed by using a calibration curve. Biomarkers were classified negative if one or both samples were below the lowest point of the calibration curve. For the miRNAs and lncRNAs (except *PCA3*), no calibration curve was used and the relative expression was calculated. For miRNAs, we used the following algorithm: If the mean Cp value of the replicates is > 37 and/or delta Cp of the replicates is > 1.0, a Cp value of 45 was used for analysis. For lncRNAs, the following algorithm was used: If the mean Cp value of the replicates is > 38 and/or delta Cp of the replicates is > 1.5, a Cp value of 45 was used for analysis. The relative RNA expression levels in mCRPC patients compared with healthy controls (using the ΔΔ*C_t_* method) were calculated. If mean baseline levels of a biomarker in patients were more than twofold higher compared with healthy controls, they were included for survival analysis. All biomarkers that did not meet this criterion were excluded for further analysis.

#### 2.4.3 Follow‐up of biomarker levels related to treatment response

To investigate whether there is a relation between biomarker levels over time and treatment response, biomarker levels were measured longitudinally. Only the biomarkers showing a relation with PFS in the univariable analyses (*P*‐value ≤ 0.1) were selected for longitudinal analyses. RNA expression levels of these biomarkers are visualized over time for patients with early progression (progression within 6 months) compared to patients with stable disease or responders (no progression within 6 months). Patients who stopped treatment due to toxicity were excluded from longitudinal analyses.

### 2.5 Pharmacokinetic assessment

The abiraterone concentration was measured by a validated liquid chromatography‐tandem mass spectrometry method [34]. Abiraterone plasma concentrations were calculated at exactly 24 h after ingestions. For calculating the trough levels, Bayesian estimation was used based on a population pharmacokinetic model described by Stuyckens *et al*. [35]. The population pharmacokinetic parameters were re‐estimated based on the data collected in our study. The average abiraterone calculated trough levels at 1, 3, and 6 months per patient were used for further analysis.

### 2.6 Statistics

The primary endpoint was a difference in PFS on first‐line AA‐P treatment explained by biomarker expression and/or drug exposure. Univariable Cox regression was used to identify whether biomarkers and drug exposure were related to PFS by estimating hazard ratios (HR) and corresponding 95% confidence intervals (CI). Cutoff values for biomarkers were calculated using the maximally selected rank statistics. The cutoff value used for the effect of abiraterone exposure on survival was the earlier identified abiraterone trough concentration of 8.4 ng·mL^−1^ [26, 27]. Pretreatment according to CHAARTED / STAMPEDE protocols and presence of visceral metastasis were added in univariable analysis too.

Biomarkers and abiraterone exposure were included in the multivariable model and Kaplan–Meier (KM) analysis if the *P*‐value was ≤ 0.1 in the univariable Cox regression. Based on a previous defined prognostic index model for PFS in chemotherapy‐naïve mCRPC patients treated with AA‐P, the following covariables were added to the multivariable model regardless of the univariable outcome: presence of lymph node metastasis, LDH > ULN, ≥ 10 bone metastasis, Hb ≤ LLN, and PSA > 39.5 ng·mL^−1^ [24]. Missing covariables were kept missing. Since this study was an exploratory study, no corrections for multiple testing were done. KM curves and log‐rank tests were used to compare differences in PFS between groups with a biomarker expression or drug exposure above and below the cutoff. Statistical significance was set at *P* < 0.05. All statistical analyses were performed in r Studio (Version 1.1.456).

## 3 Results

### 3.1 Patients

From January 2016 to October 2018, 57 patients entered the study. Four patients were excluded [two patients stopped directly, one patient stopped before the second visit, and one patient did not meet with the inclusion criteria (treated with AA‐P as second‐line treatment)]. In total, 53 patients were included for analysis. At the time of analysis, 74% of the patients (*N* = 39) showed progression, with a median PFS of 11 months (range 2–30 months) and 28% of the patients (*N* = 15) died. Radiologic progression was reported for 30 patients, biochemical progression only was reported for six patients, one patient stopped due to clinical progression and two patients died due to progressive disease. Three patients stopped AA‐P therapy because of toxicity. These patients were censored for survival analysis. The median follow‐up time for patients still on AA‐P therapy was 18 months (range 12–26 months). Baseline characteristics are summarized in Table 1.

**Table 1:** Baseline characteristics. ALP, alkaline phosphatase; DHEAS, dehydroepiandrosterone sulfate. Data are presented as median (Q1–Q3) for continuous data or *N* (%) for categorical data.

Patient characteristics at baseline	Total *N* = 53
Age at baseline (years)	71 (65–78)
Weight at baseline (kg)	86 (80–93)
Hb (mm)	7.9 (7.4–8.4)
LDH (U·L^−1^)	230 (206–264)
ALP (U·L^−1^)	85 (71–125)
Albumin (g·dL^−1^)	4.1 (3.7–4.4)
PSA (ng·mL^−1^)	39 (23–130)
PSA doubling time (months)	3.3 (2.3–6.1)
DHEAS (µm)	1.6 (0.9–2.5)
	***N* (%)**
Gleason score at diagnosis	
≤ 7	13 (24.5)
≥ 8	37 (69.8)
Missing	3 (5.7)
Ethnicity / Race	
White	53 (100)
ECOG performance status	
0	34 (64.2)
1	17 (32.1)
2	2 (3.8)
Pretreatment docetaxel^a^	16 (30.2)
Previous treatments	
Prostatectomy	20 (37.7)
Radiation	21 (39.6)
Anti‐androgen pretreatment	38 (71.7)
Other^b^	1 (1.9)
> 10 metastases at baseline	21 (39.6)
Spread of disease	
Lymph only	5 (9.4)
Bone only	14 (26.4)
Both bone and lymph	24 (45.3)
Visceral + lymph node/bone	9 (17.0)

^a^ Pretreatment with docetaxel according to CHAARTED/STAMPEDE schedule.

^b^ Tamoxifen.

### 3.2 Expression levels of circulating RNAs

Expression levels of miRNA and AR in healthy individuals were not different between the three groups of healthy controls, and therefore, their blood RNA values were pooled. *AR‐V7* was not detectable in healthy controls. All individual biomarker expression levels in healthy controls are listed in Table S8.

For miR‐375, miR‐3687, and *NAALADL2‐AS2,* the mean relative circulating RNA levels were more than twofold higher in patients compared with healthy controls and were therefore included as putative prognostic biomarkers in the survival analyses. *AR‐V7* could be detected in 6 patients at baseline and *KLK3* was detectable in 17 patients at baseline. Both were included for survival analysis. For miR‐21, miR‐141, miR‐200a, miR‐200c, *AR*, *AC012531.25,* and *SNHG3* the mean baseline levels were less than twofold higher compared with healthy controls, and therefore, no survival analysis was performed for these biomarkers. *PCA3* was detectable in only two patients, and *SCHLAP1* was not detectable in any patient and therefore excluded for survival analysis. The mean biomarker expression levels of all patients per timepoint are shown in Table 2.

**Table 2:** RNA expression levels at four timepoints. ND, not detectable.

RNA expression levels	Baseline, *N* = 53	1 month, *N* = 53	3 months, *N* = 48	6 months, *N* = 39
mRNAs				
*AR* ^a^	0.87 (0.19–2.01)	0.80 (0.09–1.88)	0.75 (0.19–1.72)	0.837 (0.22–2.45)
*AR‐V7* ^b^	6 (11.32)	2 (3.77)	1 (1.89)	2 (3.77)
*KLK3* ^b^	17 (32.08)	7 (13.21)	5 (9.43)	3 (5.66)
miRNAs^a^				
miR‐21	1.13 (0.31–4.28)	1.29 (0.37–3.67)	1.38 (0.38–3.65)	1.26 (0.23–3.03)
miR‐141	0.58 (0.00–2.00)	0.70 (0.00–3.13)	0.86 (0.00–3.16)	0.75 (0.00–2.44)
miR‐200a	0.35 (0.00–1.22)	0.31 (0.00–1.11)	0.39 (0.00–1.27)	0.53 (0.00–1.91)
miR‐200c	1.47 (0.31–3.55)	1.29 (0.13–4.06)	1.27 (0.51–3.44)	1.58 (0.55–3.33)
miR‐375	3.97 (0.46–38.43)	1.44 (0.41–25.13)	1.37 (0.65–29.25)	1.75 (0.41–5.77)
miR‐3687	3.92 (0.21–113.10)	0.72 (0.10–104.85)	1.52 (0.13–15.79)	2.81 (0.00–33.00)
lncRNAs^c^				
*PCA3* ^b^	2 (3.77)	0	0	0
*AC012531.25* ^a^	0.81 (0.00–5.96)	0.57 (0.00–3.33)	0.58 (0.00–3.26)	0.64 (0.00–5.45)
*NAALADL2‐AS2* ^a^	6.04 (0.00–79.67)	7.26 (0.00–97.74)	6.82 (0.00–78.84)	8.64 (0.00–107.33)
*SNHG3* ^a^	0.58 (0.20–1.29)	0.55 (0.22–0.91)	0.54 (0.18–0.85)	0.56 (0.24–1.00)
*SCHLAP1* ^a^	ND	ND	ND	ND

^a^ Relative expression levels compared with healthy controls, mean (range).

^b^ Number of detectable samples (≥ the lowest point of the calibration curve), *N* (%).

^c^ For the lncRNAs only samples of 52 patients were available at baseline.

### 3.3 Progression‐free survival analysis

#### 3.3.1 Univariable and multivariable Cox regression

*AR‐V7, KLK3*, miR‐375, miR‐3687, *NAALADL2‐AS2,* drug exposure, pretreatment according to CHAARTED / STAMPEDE protocols and presence of visceral metastasis were included in univariable Cox regression (Table 3). *KLK3* and *AR‐V7* were only detectable in a subset of patients, and therefore, the cutoff was set at detectable yes or no. The cutoff values for miR‐375, miR‐3687, and *NAALADL2‐AS2* calculated with maximally selected rank statistics were 2.16, 0.29, and 3.66 (relative to healthy controls), respectively.

**Table 3:** Cox regression analysis in relation to PFS.

	Univariable		Multivariable (*N* = 51^a^)	
	HR (95%CI)	*P*‐value	HR (95%CI)	*P*‐value
*AR‐V7* positive	1.37 (0.53–3.57)	0.52	–	–
*KLK3* positive	3.16 (1.59–6.27)	< 0.0001	5.07 (1.81–14.18)	0.0020
miR‐375 > cutoff	1.78 (0.93–3.41)	0.08	1.10 (0.49–2.48)	0.81
miR‐3687 > cutoff	2.41 (0.73–7.99)	0.15	–	–
*NAALADL2‐AS2* > cutoff	0.60 (0.31–1.15)	0.14	–	–
Abiraterone *C*_trough_ > 8.4 ng·mL^−1^	0.85 (0.37–1.95)	0.70	–	–
Pretreatment according to CHAARTED/STAMPEDE	0.91 (0.46–1.80)	0.78	–	–
Visceral metastasis	0.87 (0.39–1.90)	0.72	–	–
Lymph node metastasis	1.86 (0.94–3.65)	0.07	2.57 (1.20–5.50)	0.0149
LDH > ULN	1.53 (0.79–2.96)	0.21	1.77 (0.77–4.05)	0.18
Hb ≤ LLN	1.55 (0.78–3.10)	0.21	1.44 (0.63–3.33)	0.39
PSA > 39.5 ng·mL^−1^	2.32 (1.21–4.44)	0.01	1.54 (0.70–3.38)	0.29
≥ 10 bone metastases	2.39 (1.23–4.64)	0.0098	1.62 (0.72–3.62)	0.24

^a^ Two observations deleted due to missing covariables.

High levels of miR‐375 and detectable *KLK3* mRNA levels were related to PFS (HR 1.78; 95% CI 0.93–3.41; *P* = 0.08 and HR 3.16; 95% CI 1.59–6.27; *P *=< 0.0001). All other parameters were not related to PFS (in univariable analysis) and therefore not included in multivariable Cox regression analysis. In a multivariable Cox regression model, detectable *KLK3* mRNA levels and presence of lymph node metastasis were both independent predictors of shorter PFS (HR 5.07; 95% CI 1.81–14.18; *P* = 0.0020 and HR 2.57; 95% CI 1.20–5.50, *P* = 0.0149 respectively; Table 3).

#### 3.3.2 Kaplan–Meier analysis

**Fig. 1:**
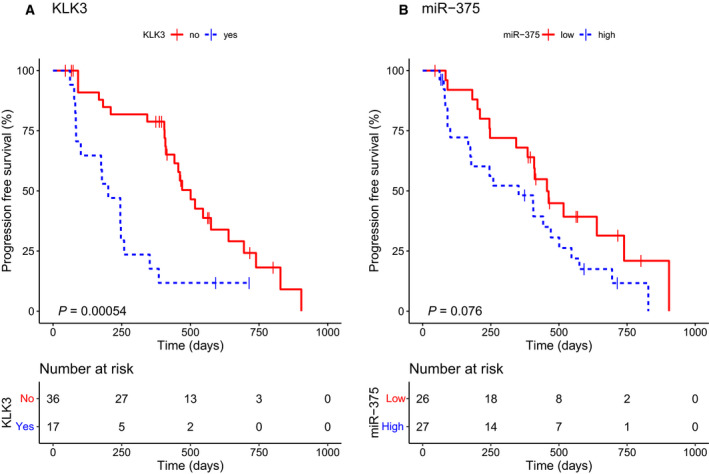
KM curves for PFS. (A) PFS according to KLK3 detection at baseline: nondetectable vs detectable as cutoff value in abiraterone‐treated patients. (B) PFS according to miR‐375 expression levels at baseline: < 2.16 vs ≥ 2.16 relative to healthy controls as cutoff value in abiraterone‐treated patients. *P*‐values are calculated by log‐rank test.

Patients with detectable levels of *KLK3* had a significantly shorter PFS compared to patients without *KLK3* detection, median 201 vs 501 days (*P* = 0.00054). Patients with expression levels of miR‐375 above cutoff showed shorter PFS compared to patients with miR‐375 levels below cutoff, although not statistically significant, median 352 vs 456 days (*P* = 0.076). Figure 1A,B shows the survival curves.

### 3.4 Abiraterone exposure

The abiraterone trough concentration (*C*_trough_) was measured in 52 patients. Pharmacokinetic assessment in one patient failed due to errors made in blood sample collection. The mean calculated abiraterone *C*_trough_ was 14.5 ng·mL^−1^ with a range of 3.4–92.1 ng·mL^−1^. The intra‐ and interpatient variability was 30.2% and 49.5%, respectively. In total, 10 patients had a mean *C*_trough_ level below the threshold of 8.4 ng·mL^−1^. In univariable Cox regression analysis a mean *C*_trough_ level ≥ 8.4 ng·mL^−1^ was not related to PFS (HR 0.85; 95% CI 0.37–1.95, *P* = 0.70; Table 3).

### 3.5 Longitudinal follow‐up of treatment response using *AR‐V7*, *KLK3,* and miR‐375

During treatment, 13 patients stopped AA‐P therapy due to progression within 6 months (early progression, median time to PFS of 3 months). Expression levels of *AR‐V7*, *KLK3,* and miR‐375 over time in patients with early progression were compared with patients who responded to treatment (stable disease or partial response, median PFS follow‐up of 15 months).

**Fig. 2:**
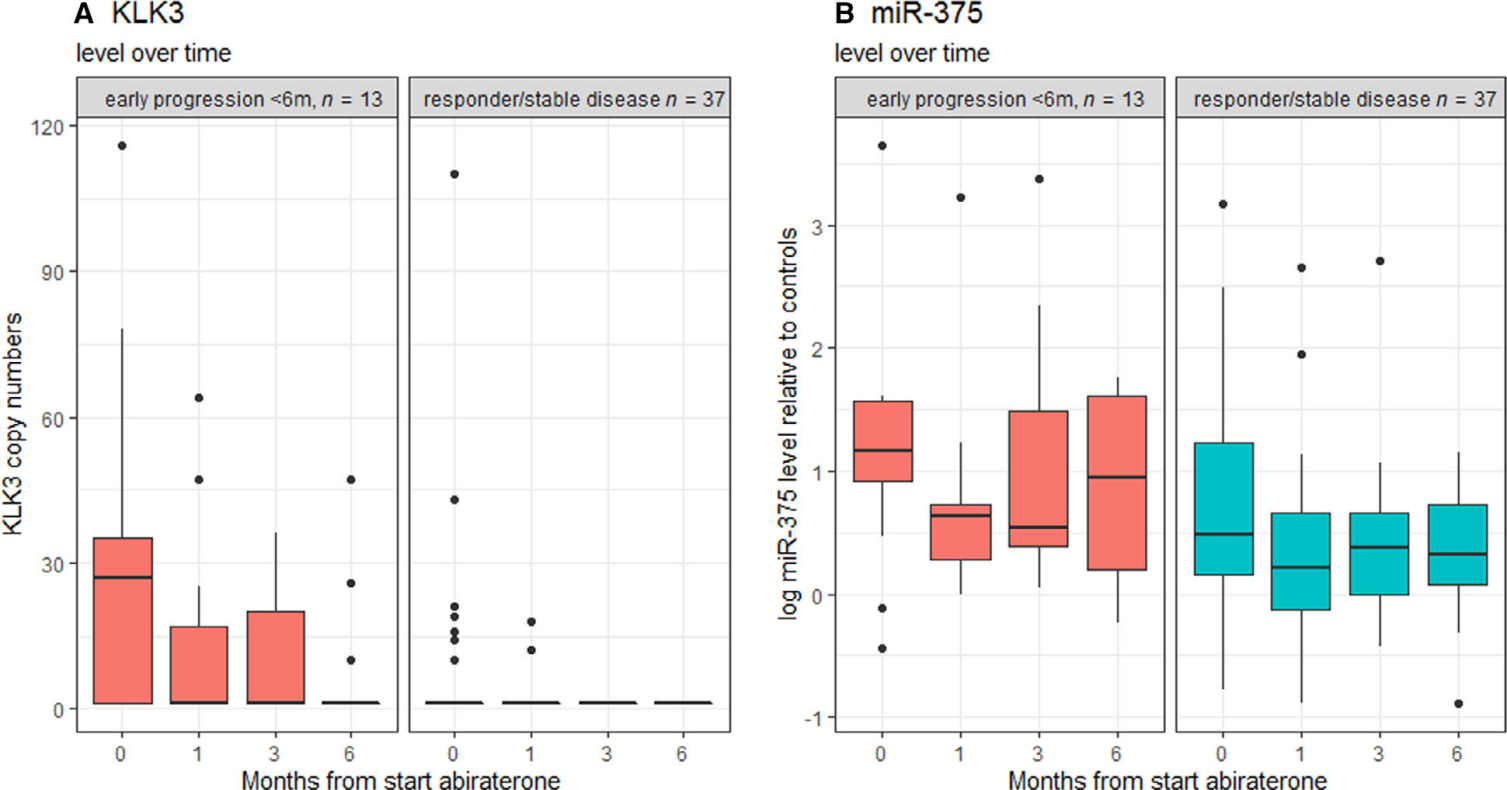
Boxplots of KLK3 (A) and miR‐375 (B) expression levels over time. Left panel shows patients who progressed early (< 6 months) after start of AA‐P therapy, right panel shows patients with stable disease / responders to AA‐P therapy (progression ≥ 6 months). The bottom and top of the box are the 25th and 75th percentiles, and the line inside the box is the median.

At baseline, six patients were *AR‐V7* positive, of which five patients responded to treatment (stable disease or partial response). During treatment, *AR‐V7* positivity dropped in all six patients to undetectable levels. Expression levels of *KLK3* are higher and more frequent detectable in patients with early progression compared with responders. Under treatment, expression levels of *KLK3* decreased. For patients who responded to AA‐P treatment, *KLK3* was not detectable in any patient at 3 months, where in patients with early progression *KLK3* was still detected in five patients (55.6%) (Fig. 2A).

At baseline, 17 patients were *KLK3* positive of which seven patients responded to treatment (stable disease or partial response) and 10 patients had early progression. At 6 months, only three patients were *KLK3* positive (Fig. 2A and Table 2). This drop is due to the fact that 8 *KLK3*‐positive patients at baseline dropped out before evaluation at 6 months due to early progression. Furthermore, in seven patients KLK3 levels dropped to undetectable levels after 3 months, which is reflecting treatment response. One patient became *KLK3* positive at 6 months during treatment. Levels of miR‐375 showed an overall decrease after start of AA‐P therapy regardless of response. Patients with early progression had higher levels of miR‐375 compared with responders, especially at baseline. However, no clear pattern in changes in miR‐375 levels over time was observed between the responders and patients with early progression (Fig. 2B).

## 4 Discussion

In this study, the prognostic value of *KLK3* mRNA and other novel circulating RNAs in first‐line mCRPC patients starting AA‐P was studied. Furthermore, the effect of abiraterone exposure on treatment outcome was evaluated. Our study confirmed that detectable levels of *KLK3* at baseline appear to be prognostic for shorter PFS. Furthermore, circulating *KLK3* mRNA levels outperform all other investigated clinical prognostic biomarkers.

To the best of our knowledge, this is the first prospective clinical trial confirming *KLK3* as a prognostic biomarker for PFS. Previous work revealed that *KLK2/3* mRNA detection in whole blood by RT–PCR is highly concordant with CTCs detected by the CellSearch system [36]. Danila *et al*. [9] investigated whether the CTCs detected with the CellSearch and AdnaTest assay or the direct detection of tumor mRNA with a ddPCR method were the best biomarker for OS. The ddPCR and AdnaTest had higher detection rates compared with the CellSearch system, and therefore were superior to the CellSearch system. Because *KLK3* mRNA detection appeared to be the most relevant marker for a positive result by the AdnaTest and ddPCR, detection of *KLK3* mRNA in whole blood can be used as a surrogate marker for CTC counts [9]. Since CTC isolation is technically and logistically challenging, the clinical utility of CTC enumeration in this disease setting is minimal. We showed that our RT–PCR method for *KLK3* detection in whole blood collected in PAXgene tubes is a sensitive method and could be used in first‐line patients. The method requires minimal sample pretreatment and therefore can easily be implemented into clinical practice. *KLK3* mRNA detection in early disease setting, but presumably also in patients with more advanced disease, might therefore be an easier to measure biomarker compared to CTC detection.

By longitudinally follow‐up of *KLK3* mRNA levels, we assessed the potential value for monitoring treatment response. Previous work in CRPC patients treated with docetaxel revealed that *KLK3* levels in patients responding to docetaxel were decreased [7]. In our study, we found similar results, all patients responding to AA‐P (median PFS follow‐up of 15 months) had undetectable *KLK3* after 3 months of treatment. Because *KLK3* is the transcript encoding for PSA, one might think that *KLK3* would be related to PSA levels. However, in our multivariable Cox regression analysis serum PSA was not prognostic for survival. This emphasizes the additional value of *KLK3* mRNA detection over serum PSA measurement. Although serum PSA is clinically used to monitor treatment response, serum PSA levels may not reflect the status of the disease accurately [37]. CTC enumeration has been incorporated by the PCWG3 as a clinical endpoint in trials for treatment response [38]. Since *KLK3* mRNA detection likely reflects CTC burden, *KLK3* detection may alternatively be used to assess treatment response. This hypothesis should be confirmed in a larger well‐annotated patient cohort with time to progression data and blood collection for translational RNA studies.

Another potentially promising RNA biomarker is miR‐375. However, in multivariable analysis and KM analysis, no statistically significant effect was seen in our cohort which might be due to our limited number of patients. Several previous studies showed that higher miR‐375 levels at baseline are related to shorter survival [13, 39]. Therefore, miR‐375 is potentially promising and should be considered for preclinical functionality testing and future clinical validation.

MiR‐3687 and *NAALADL2‐AS2* were also upregulated in PCa patients compared with healthy controls. However, both biomarkers failed to be related to PFS. In our previous cohort of first‐line mCRPC patients treated with enzalutamide, high levels of miR‐3687 and low levels of *NAALADL2‐AS2* were significantly prognostic of shorter PFS. Since the functionality of miR‐3687 and *NAALADL2‐AS2* in PCa is currently not clear, preclinical functionality testing is required before these results can be interpreted. Future clinical validation of these biomarkers in a larger independent cohort is needed.

In our study, *AR‐V7* RNA levels were not prognostic for survival. Our study included only first‐line AA‐P patients. Six out of 53 patients (11.3%) were *AR‐V7* positive with copy numbers just above the lower limit of quantification, while AR levels were not distinctive from healthy volunteers. A previous study showed that only 3% of first‐line patients were *AR‐V7* positive [23]. In our study, a whole blood *AR‐V7* test is used, while most studies have been performed with *AR‐V7* detection in CTCs. *AR‐V7* detection in whole blood might have a lower sensitivity compared with CTC‐based *AR‐V7* detection. On the other hand, many first‐line patients are CTCs negative, and therefore, CTC‐based *AR‐V7* detection is not useful. It has been shown that whole blood *AR‐V7* positivity is correlated with CTC counts and that patients with undetectable CTC levels still can be *AR‐V7* positive when using whole blood [40]. Therefore, the predictive value of *AR‐V7* in whole blood might only be clinically relevant for higher copy numbers of *AR‐V7* which can be detected at later stages of treatment. Future research should compare CTC‐based and whole blood‐based *AR‐V7* detection, and determine a clinically relevant threshold for *AR‐V7* detection in whole blood. Next to the biomarkers, we studied the effect of drug exposure on PFS. It has been suggested that underexposure of abiraterone (*C*_trough_ level < 8.4 ng·mL^−1^) is related to shorter PFS [26, 27]. The beneficial effect of higher abiraterone exposure levels could not be confirmed in this study for chemotherapy naïve patients treated with AA‐P. This could be due to our study cohort of only first‐line patients. Xu *et al*. [41] found that the effective concentration levels of abiraterone in chemotherapy‐naïve patients were lower compared with postchemotherapy patients. The previously defined threshold has been established in a mixed cohort of chemotherapy‐naïve and chemotherapy‐pretreated patients. Future research is necessary to investigate the threshold for abiraterone in chemotherapy‐naïve patients treated with AA‐P.

In our study, besides *KLK3* mRNA, the prognostic value of the other biomarkers and drug exposure could not be confirmed. Since the prostate landscape is changing, and the indication for drugs such as AA‐P has broadened from the castration‐resistant to the hormone‐sensitive setting, it is important to study the value of biomarkers and drug exposure in a clear subset of patients. In this light, we set up a well‐defined cohort of only first‐line mCRPC patients treated with AA‐P. This explains the lower expression levels of most RNAs compared with previous studies and underscores the need to investigate the added value of biomarkers in well‐defined stage of disease.

Although the biomarker research field is growing, there remains a large gap between biomarker discovery and clinical validation. Therefore, prospective clinical validation of biomarkers is needed. Our study is a step forward in biomarker validation, providing valuable information in an independent group of patients in a prospective study design about the clinical validation of *KLK3* mRNA and absent value of the other investigated biomarkers in identifying patients who will benefit shorter from AA‐P therapy. A strength of our study is that we have used healthy controls to make sure that the biomarkers we measured are related to prostate cancer. Only biomarkers which were upregulated in CRPC patients compared with healthy controls are selected. Therefore, our working hypothesis is that the identified whole blood RNA biomarkers are reflecting disease burden. Expression levels of many of the circulating RNAs in our cohort were not distinctive from expression levels in healthy controls. This highlights the need of incorporating a control cohort to identify biomarkers that are specifically upregulated in patients with mCRPC. Results of biomarker studies without healthy volunteers should therefore be interpreted with more caution.

## 5 Conclusions

Our study confirmed *KLK3* mRNA as an independent prognostic marker for PFS in mCRPC patients receiving first‐line AA‐P treatment. Detection of whole blood *KLK3* could be easily incorporated into clinical practice. *KLK3* detection over time may be a potential biomarker to monitor treatment response or progression, but prospective clinical validation is needed.

## Conflict of interest

JAS and GWV are inventors on the PCA3‐related IP. The IP is owned by their employer, Radboud university medical center, which has licensed the technology and receives royalty payments. IMvO: Astellas, Janssen‐Cilag, Bayer, SelectMDx, Roche; NPHvE: Astellas, Janssen‐Cilag, Sanofi, Bayer; N.M.: Advisory role (compensated and institutional): Roche, MSD, BMS, Bayer, Astellas and Janssen’. Research support (institutional): ‘Astellas, Janssen, Pfizer, Roche and Sanofi’ Genzyme. Travel support: ‘Astellas, MSD’. All remaining authors have declared no conflicts of interest.

## Author contributions

EB and GEB managed the project, analyzed the data, and wrote the manuscript with input from all authors. JAS and NPE designed the study. EB, GEB, IvMO, IMO, PM, TJS, DMS, and NM were responsible for patient inclusion. GWV, OH, LG, and FS performed the experiments. GWV, JAS, NPE, IvMO, and NM supervised the project. All authors contributed to the interpretation of the data, discussed the results, contributed to the manuscript, and approved the final version.

### Peer Review

The peer review history for this article is available at https://publons.com/publon/10.1002/1878‐0261.12933.

## Supporting information

**Table S1**. Sequences of synthetic *C. elegans* miRNAs.
**Table S2**. Sequences and amplicon size of primer pairs used for PCR analysis.
**Table S3**. Sequences of hydrolysis probes for qPCR analysis.
**Table S4**. Stem‐loop reverse transcriptase (SL‐RT) primers.
**Table S5**. Sequences of primer pairs used for miRNA PCR analysis.
**Table S6**. RT reaction conditions.
**Table S7**. PCR cycle conditions.

**Table S8**. Overview of biomarker expression levels in healthy controls.

## Data Availability

The data that support the findings of this study are available in the [Link], [Link] of this article and from the corresponding author upon reasonable request.
