# Supplementary material for: Liquid biopsy reveals KLK3 mRNA as a prognostic marker for progression free survival in patients with metastatic castration‐resistant prostate cancer undergoing first‐line abiraterone acetate and prednisone treatment
Source: Mol Oncol. 2021 May 29;15(9):2453–65. doi: 10.1002/1878-0261.12933 (PMC8410566; doi:10.1002/1878-0261.12933)
Supplement: Supplementary file 1 — Table S1. Sequences of synthetic C. elegans miRNAs. Table S2. Sequences and amplicon size of primer pairs used for PCR analysis. Table S3. Sequences of hydrolysis probes for qPCR analysis. Table S4. Stem‐loop reverse transcriptase (SL‐RT) primers. Table S5. Sequences of primer pairs used for miRNA PCR analysis. Table S6. RT reaction conditions. Table S7. PCR cycle conditions. [file MOL2-15-2453-s002.docx]

**Supplementary Table 1: Sequences of synthetic *C.elegans* miRNAs**

| **Gene** | **Sequence 5’ 🡪 3’** | **Manufacturer** |
| --- | --- | --- |
| *C.el* miR-39 | UCACCGGGUGUAAAUCAGCUUG | Invitrogen (ThermoFisher) |
| *C.el* miR-238 | UUUGUACUCCGAUGCCAUUCAGA | Invitrogen (ThermoFisher) |

**Supplementary Table 2: Sequences and amplicon size of primer pairs used for PCR analysis**

| **Gene** | **Forward primer (5’ 🡪 3’)** | **Reverse primer (5’ 🡪 3’)** | **Size (bps)** |
| --- | --- | --- | --- |
| *AC012531.25 (AC2)* | CCCTGTGGTGGACAGACTTC | GGGTGGGCTCAACTTTCTCA | 113 |
| *AR* (full-length) | AAGGAACTCGATCGTATCATTGC | TTGGGCACTTGCACAGAGAT | 209 |
| *AR-v7* | TGTCGTCTTCGGAAATGTTATGA | TCATTTTGAGATGCTTGCAATTG | 82 |
| *GAPDH* | TCAAGGCTGAGAACGGGAAG | TGGACTCCACGACGTACTCA | 117 |
| *KLK3* | CGTGACGTGGATTGGTGC | ACTGCCCTGCCACGAGAG | 113 |
| *NAALADL2-AS2* | ACCAGCGGAAATTGAAAAGCAA | GCTCAGCTCATGTCTTCTCCT | 124 |
| *PCA3* | GAAGCTGGCATCAGAAAAACAGAG | AGATGTGTGGCCTCAGATGGTAAA | 310 |
| *SCHLAP1* | TGGACACAATTTCAAGTCCTCA | CATGGTGAAAGTGCCTTATACA | 88 |
| *SNHG3* | GATGGTAGCAACGGGAGGTT | ATGATCCCCCTTCAGTCCCA | 196 |

**Supplementary Table 3: Sequences of hydrolysis probes for qPCR analysis**

| **miRNA** | **Probe sequence (5’ 🡪 3’)** | **Fluorophore** |
| --- | --- | --- |
| *AR* (full-length) | TGATGCAGCTCTCTCGCAATAGGC | FAM |
| *AR-v7* | TTTCTCCCAGAGTCATCCCTGCT | HEX |
| *KLK3* | CCCTCATCCTGTCTCGGATTGTGGGA | HEX |
| *PCA3* | TGCATGGTGGGAAGGACCTGATGA | FAM |

**Supplementary Table 4: Stem-loop reverse transcriptase (SL-RT) primers**

| **miRNA** | **SL-RT primer sequence (5’ 🡪 3’)** |
| --- | --- |
| RNU6-1 | GTCATCCTTGCGCAGG |
| miR-21 | GTCGTATCCAGTGCAGGGTCCGAGGTATTCGCACTGGATACGACTCAACA |
| miR-141 | GTCGTATCCAGTGCAGGGTCCGAGGTATTCGCACTGGATACGACCCATCT |
| miR-200a | GTCGTATCCAGTGCAGGGTCCGAGGTATTCGCACTGGATACGACACATCG |
| miR-200c | GTCGTATCCAGTGCAGGGTCCGAGGTATTCGCACTGGATACGACTCCATC |
| miR-375 | GTCGTATCCAGTGCAGGGTCCGAGGTATTCGCACTGGATACGACTCACGC |
| miR-3687 | CTCGTATCCAGTGCAGGGTCCGAGGTATTCGCACTGGATACGAGACGTCG |
| *C.el* miR-39 | GTCGTATCCAGTGCAGGGTCCGAGGTATTCGCACTGGATACGACCAAGCT |
| *C.el* miR-238 | GTCGTATCCAGTGCAGGGTCCGAGGTATTCGCACTGGATACGACTCTGAA |

**Supplementary Table 5: Sequences of primer pairs used for miRNA PCR analysis**

| **miRNA** | **Forward (5’ 🡪 3’)** | **Reverse (5’ 🡪 3’)** |
| --- | --- | --- |
| RNU6-1 | CGCTTCGGCAGCACATATAC | AGGGGCCATGCTAATCTTCT |
| miR-21-5p | GCCCGCTAGCTTATCAGACTGATG | GTGCAGGGTCCGAGGT  (universal reverse primer) |
| miR-141-3p | GCCCGCTAACACTGTCTGGTAAAG |  |
| miR-200a-3p | GCCCGCTAACACTGTCTGGTAACG |  |
| miR-200c-3p | GCCCGCTAATACTGCCGGGTAATG |  |
| miR-375-3p | TGCCAGTTTGTTCGTTCGGCTC |  |
| miR-3687 | TGCAAGCCCGGACAGGCGTTCGTG |  |
| *C.el* miR-39 (-3p) | TGCCAGTCACCGGGTGTAAATC |  |
| *C.el* miR-238 (-3p) | TGCCAGTTTGTACTCCGATGCCA |  |

| **RNA** | **RT primer** | **RT reaction** | **Reverse Transcriptase** | **Supplier** |
| --- | --- | --- | --- | --- |
| *KLK3, PCA3* | Target-specific | 42^o^C/30 min. | QuantiScript RT | Qiagen |
| *AR, AR-V7* | Target-specific | 50^o^C/10 min. | Fast virus One-step RT-PCR | Applied biosystems |
| MicroRNAs | Target-specific | 16^o^C/30 min. 42^o^C/30 min.  85^o^C/5 min. | SuperScript II RT | Invitrogen |
| LncRNAs | Hexamer random-primed | 40^o^C/60 min. | SuperScript II RT | Invitrogen |

**Supplementary Table 6: RT reaction conditions**

**Supplementary Table 7: PCR cycle conditions**

| **Gene product** | **Initial denaturation** | **Cycle program** | **No. Cycles** | **Melt Curve analysis** |
| --- | --- | --- | --- | --- |
| *KLK3, PCA3* | 95^o^C/10 min. | 95^o^C/10 sec., 60^o^C/30 sec. | 50 | no |
| *AR, AR-V7* | 95^o^C/30 sec. | 95^o^C/5 sec., 60^o^C/30 sec. | 50 | no |
| MicroRNAs | 95^o^C/5 min. | 95^o^C/10 sec., 60^o^C/20 sec., 72^o^C/10 sec. | 45 | yes |
| LncRNAs | 95^o^C/5 min. | 95^o^C/10 sec., 60^o^C/20 sec., 72^o^C/20 sec. | 45 | yes |
