# Supplementary material for: Liquid biopsy reveals KLK3 mRNA as a prognostic marker for progression free survival in patients with metastatic castration‐resistant prostate cancer undergoing first‐line abiraterone acetate and prednisone treatment
Source: Mol Oncol. 2021 May 29;15(9):2453–65. doi: 10.1002/1878-0261.12933 (PMC8410566; doi:10.1002/1878-0261.12933)
Supplement: Supplementary file 2 — Table S8. Overview of biomarker expression levels in healthy controls. [file MOL2-15-2453-s001.docx]

|  | **MicroRNAs (Cp-values)** | | | | | | **Long non-coding RNAs (Cp-values)** | | | | **mRNAs (copies/ug RNA)** | |
| --- | --- | --- | --- | --- | --- | --- | --- | --- | --- | --- | --- | --- |
|  | **miR-21** | **miR-141** | **miR-200a** | **miR-200c** | **miR-375** | **miR-3687** | **AC012531.25** | **SNHG3** | **NAALADL2-AS2** | **SCHLAP1** | **ARv7** | **ARFL** |
| **Men (≤35 y/o)** | 22.09 | 33.88 | 33.02 | 26.25 | 31.03 | 32.06 |  |  |  |  | ND | 4343 |
| **Men (≤35 y/o)** | 23.03 | 34.87 | 34.27 | 27.08 | 31.48 | 30.97 |  |  |  |  | ND | 4000 |
| **Men (≤35 y/o)** | 22.65 | 35.56 | 34.44 | 26.51 | 31.08 | 32.07 |  |  |  |  | ND | 3257 |
| **Men (≤35 y/o)** | 23.01 | 36.41 | 40.00 | 26.92 | 31.14 | 31.30 |  |  |  |  | ND | 2700 |
| **Men (≤35 y/o)** | 21.56 | 34.21 | 33.72 | 26.54 | 31.24 | 31.61 |  |  |  |  | ND | 4000 |
| **Men (≤35 y/o)** | 23.07 | 35.58 | 34.55 | 26.92 | 31.85 | 32.24 |  |  |  |  | ND | 4514 |
| **Men (≤35 y/o)** | 21.90 | 33.58 | 33.51 | 26.65 | 30.73 | 31.06 |  |  |  |  | ND | 5371 |
| **Men (≤35 y/o)** | 23.03 | 35.05 | 34.58 | 26.81 | 31.68 | 31.86 |  |  |  |  | ND | 4629 |
| **Men (≤35 y/o)** | 23.36 | 36.81 | 34.82 | 26.71 | 31.34 | 32.20 |  |  |  |  | ND | 4114 |
| **Men (≤35 y/o)** | 21.82 | 34.99 | 35.45 | 26.43 | 30.66 | 31.79 |  |  |  |  | ND | 3229 |
| **Men (53-70 y/o)** | 21.94 | 34.26 | 37.01 | 26.53 | 30.80 | 31.89 | 36.92 | 26.66 | 35.59 | ND | ND | 2943 |
| **Men (53-70 y/o)** | 22.68 | 33.34 | 33.99 | 26.94 | 32.30 | 31.88 | 37.06 | 25.64 | 32.06 | ND | ND | 2626 |
| **Men (53-70 y/o)** | 20.74 | 33.01 | 33.83 | 26.47 | 31.86 | 31.56 | 40.00 | 26.00 | 35.27 | ND | ND | 4743 |
| **Men (53-70 y/o)** | 21.67 | 34.22 | 33.83 | 26.30 | 31.46 | 31.02 | 40.00 | 26.05 | 35.12 | ND | ND | 4943 |
| **Men (53-70 y/o)** | 21.79 | 33.84 | 33.86 | 26.46 | 31.29 | 31.01 | 36.49 | 25.77 | 32.37 | ND | ND | 7914 |
| **Men (53-70 y/o)** | 23.00 | 35.95 | 36.75 | 27.08 | 32.47 | 31.20 | 36.14 | 25.93 | 32.88 | 40.00 | ND | 2731 |
| **Men (53-70 y/o)** | 21.50 | 34.61 | 34.48 | 26.54 | 31.50 | 31.54 | 38.44 | 25.78 | 34.38 | ND | ND | 2886 |
| **Men (53-70 y/o)** | 23.83 | 36.09 | 34.73 | 26.98 | 31.90 | 31.05 | 40.00 | 26.31 | 33.84 | 20.89 | ND | 3229 |
| **Men (53-70 y/o)** | 21.70 | 33.51 | 33.30 | 27.48 | 32.74 | 30.57 | 36.10 | 25.49 | 32.03 | 40.00 | ND | 2971 |
| **Men (53-70 y/o)** | 24.61 | 36.40 | 37.09 | 27.67 | 32.21 | 30.68 | 35.43 | 25.66 | 33.18 | 21.15 | ND | 4857 |
| **Female** | 23.07 | 33.81 | 33.44 | 26.77 | 30.77 | 28.03 |  |  |  |  | ND | 2340 |
| **Female** | 21.04 | 33.29 | 33.77 | 26.33 | 30.68 | 31.21 |  |  |  |  | ND | 3057 |
| **Female** | 20.62 | 32.89 | 32.63 | 25.75 | 29.99 | 30.83 |  |  |  |  | ND | 3571 |
| **Female** | 22.88 | 35.74 | 34.99 | 27.54 | 31.68 | 31.43 |  |  |  |  | ND | 3371 |
| **Female** | 21.94 | 35.45 | 34.50 | 26.18 | 31.57 | 31.74 |  |  |  |  | ND | 3543 |
| **Female** | 21.61 | 34.12 | 33.86 | 26.31 | 30.82 | 31.07 |  |  |  |  | ND | 6229 |
| **Female** | 20.89 | 32.73 | 33.09 | 25.83 | 29.71 | 30.84 |  |  |  |  | ND | 4629 |
| **Female** | 21.10 | 33.35 | 33.68 | 26.31 | 30.95 | 31.32 |  |  |  |  | ND | 4057 |
| **Female** | 21.08 | 34.64 | 33.97 | 26.26 | 31.43 | 31.18 |  |  |  |  | ND | 3057 |
| **Female** | 20.65 | 31.79 | 33.92 | 25.92 | 31.03 | 31.11 |  |  |  |  | ND | 2783 |

**Supplementary Table 8. Overview of biomarker expression levels in healthy controls**

ND; non-detectable
